# "Signet-ring" cell invasive lobular carcinoma of the breast – accidental finding associated with intraductal papilloma: a case report

**DOI:** 10.1186/1757-1626-2-130

**Published:** 2009-02-06

**Authors:** Valerija Blažičević, Blaženka Staklenac, Jozo Kristek, Marija Pajtler, Zlatko Krajinović, Damir Štimac, Zdravko Ivezić, Zdenka Kotromanović, Ilijan Tomaš, Marta Biljan

**Affiliations:** 1 Department of Pathology and Forensic Medicine, Clinical Hospital Osijek, Osijek, Croatia; 2 Department of Clinical Cytology, Clinical Hospital Osijek, Osijek, Croatia; 3 Department of Surgery, Clinical Hospital Osijek, Osijek, Croatia; 4 Department of Radiology, Clinical Hospital Osijek, Osijek, Croatia; 5 Department of Oncology, Clinical Hospital Osijek, Osijek, Croatia

## Abstract

**Background:**

A 57-year old woman had only unilateral milky dischardge of the right breast. Clinical and mammography findings were normal.

**Case presentation:**

Cytological diagnosis of intraductal papilloma was established which was galactographically confitmed and patient underwent to surgery. Ductulolobular segmentectomy was made. Histopathologically beside intraductal papilloma numerous single dispread malignant "signet ring" cells in the fibrous retromammilary stroma were found. Imunohistochemically findings were: cytokeratin 8 positive, ER H-score 80, PR H-score 50, HER-2/neu negative. Diagnosis of "signet ring" cell lobular invasive carcinoma was made, followed by mastectomy, axillary limphadectomy and contra lateral breast biopsy.

**Conclusion:**

Residual tumor were found only in the breast tissue, while axillary lymph nodes and contra lateral breast biopsy were negative. Patient underwent to oncology therapy.

## Background

The term lobular carcinoma was established in 1941 with publication of the classic paper on this carcinoma by Foot and Stewart [1].

Invasive lobular carcinoma usually constitutes 3–5% of the invasive carcinomas, when the diagnosis is based strictly on the criteria of Foot and Stewart [2].

The WHO classification of breast tumors states that invasive lobular carcinoma is composed of uniform cells resembling those of lobular carcinoma in situ and usually having a low mitotic rate [3]. „The cells grow typically in a single-file, linear arrangement, or appear individually embedded in fibrous tissue. Infiltrating cells are often arranged concentrically around ducts, in a target-like pattern. Identification of remnants of lobular carcinoma in situ aids in the diagnosis. Tumor cells may appear in signet ring shapes owing to distension with mucus.“ Invasive lobular carcinoma occurs throughout most of the age range of the breast carcinoma in adult women (28 to 86 years). It is relatively more common among women over 75 years old (11%) than in women 35 years old or younger [4]. The presenting symptom in almost all cases is a mass with ill – defined margin. In some patients, the only evidence of the neoplasm is vague thickening or fine diffuse nodularity of the breast. Infiltrating lobular carcinoma does not have a specific or characteristic clinical and mammography appearance.

## Case presentation

### Clinical history

A fifty-seven-year-old woman had only unilateral milky discharge of the right breast. Clinical and mammography findings were normal. Ultrasound examination was performed with 10 MHz linear probe, and it showed homogeneous predominantly fatty parenchyma echotexture with no masses, cystic formations or calcifications visualised. No biopsy nor FNA was recommended, and the sonography finding was reported as BI-RADS 1. Cytological diagnosis of intraductal papilloma was established which was galactographically confirmed and patient underwent to surgery. Ductulolobular segmentectomy was made. After the histopathology results were received, besides the intraductal papilloma, there was found „signet-ring“ cell lobular invasive carcinoma. It was followed by total mastectomy, axillary limphadectomy and contra lateral breast biopsy. Residual tumor was found only in breast tissue, while axillary lymph nodes and contra lateral breast biopsy were negative.

Post-operative oncology therapy was instituted.

Review 12 months post-operatively revealed no evidence of recurrence of the tumor.

### Cytological findings

**Figure 1:**
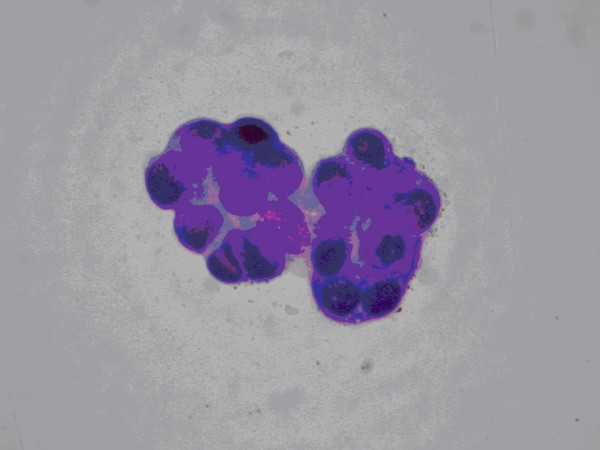
**Papillary groups of intraductal cells (MGG ×1000)**.

Milky nipple secretion of middle canal was spread to the slide, air dried and stained according to May – Grunewald – Giemsa. Cytological examination showed foam cells and cell clusters with papillary architecture, moderately enlarged nuclei of variable sizes with mild hyperchromasia. Abundant, dense, well defined cytoplasm with large, prominent vacuoles was also found. Cytological diagnosis of intraductal papilloma was established and galactography was recommended. (Figure 1)

### Histopathology

The excised specimen was not visibly abnormal and was only slightly firm to palpation although substantial involvement by tumor was evident microscopically.

Dilated mammary duct was containing solitary discrete benign papillary epithelial tumor which was consisted of an orderly proliferation of ductal epithelium on well defined fibro vascular stalks. The epithelium, distributed in a single layer, had little cellular polymorphism, and a myoepithelial layer was present between the fibro vascular stalk and the epithelial cells.

Carcinoma was not nodular, it consisted of solitary tumor cells spread throughout the whole breast parenchyma. Tumor cells existed beyond the segment of intraductal papilloma.

**Figure 2:**
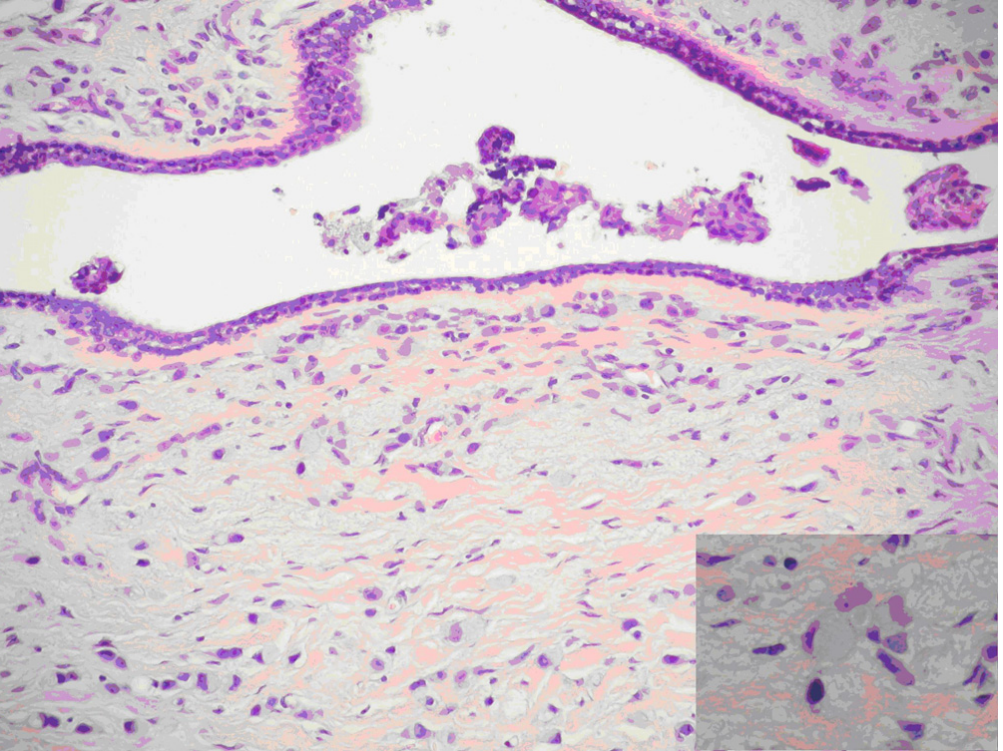
**Tissue section: Signet ring cell carcinoma**. The invasive cells assume a lobular growth pattern(HE × 100) INSET: the cells contain abundant intracytoplasmic mucin conferring a signet ring cell appearance to the cells (HE × 400)

Around dilated duct the tumor cells were arranged in concentric rings but loosely dispersed throughout a fibrous stroma, too. Most tumor cells had intracytoplasmic lumina containing sialomucins demonstrable with the mucicarmine and Alcian blue stains, and the cells had a signet ring configuration (Figure 2)

### Imunohistochemistry

**Figure 3:**
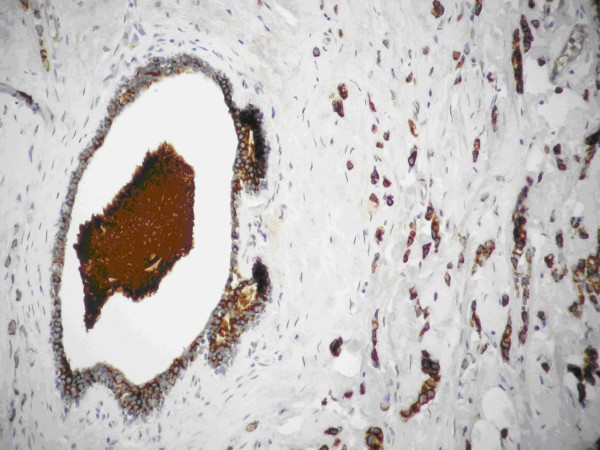
**Tissue section: the most invasive cells are imunoreactive to cytokeratin 8 (IHC ×400)**.

Further analysis was performed using the streptavidin – biotin – imunoperoxidase technique. The tumor cells were diffusely positive for Cytokeratin 8 (Figure 3) ER H-score 80, PR H-score 50, HER-2/neu negative, Ki 67 1, 5%.

## Discussion

Classic invasive lobular carcinoma is usually described as consisting of small, uniform cells with round nuclei and inconspicuous nucleoli. A variable proportion of cells have intracytoplasmic lumina containing sialomucins demonstrable with the mucicarmine and Alcian blue stains [5,6]. When the secretion is prominent, the cells have a signet ring configuration. Most so-called signet ring cell carcinomas are forms of invasive lobular carcinoma [[5-7], and [8]].

Intraductal papilloma is a discrete benign papillary epithelial tumor that arises in the mammary ducts. Although most common in subareolar lactiferous ducts, they may occur elsewhere in the breast, occasionally in cystically dilated ducts.

Solitary subareolar or intracystic intraductal papillomas are benign; however, for many years there has been a debate as to whether they may be precursors of papillary carcinoma, or whether they predispose the breast to an increased risk of carcinoma. Most follow-up studies have failed to demonstrate an increased carcinoma risk associated with these lesions.

Solitary papillomas lack implication for an increased risk of subsequent carcinoma in the remainder of the breast and may be safely treated by local excision [9].

On the other hand, multiple intraductal papillomas, which produce a mass in the periphery of the breast, are more often associated with concurrent or subsequent carcinoma. Two of the six patients reported by Carter developed carcinoma, and a similar incidence of associated carcinoma has been noted by others [10,11].

Fine-needle aspiration cytological examination of papillary lesions is increasingly performed, especially for intracystic lesions in the periphery of the breast. The procedure holds little value for subareolar papillomas which should be surgically excised and assessed by conventional histopathology techniques.

Cytological examination of material from a nipple discharge may be diagnostic if carcinoma cells are identified, but cannot be relied upon to rule out carcinoma if the cellular composition is benign [12].

Since infiltrating lobular breast carcinoma is less common than infiltrating ductal carcinoma, especially the variant with signet ring cells, and since its clinical presentation, mammography and ultrasound appearance are non-specific (meaning that they can be overseen even in the later stages of the disease), only multidisciplinary approach to the treatment of the slightest symptoms of the breast lesions can help establish the correct diagnosis.

Spontaneous breast discharge can present the only symptom (when palpable and non-palpable lesions for aspiration biopsy and histopathologycal examination are absent); therefore, exfoliative cytodiagnostic emerges as an inevitable and relevant method in explaining its etiology [13-18]. However, because of its high frequency, the breast discharge is a serious problem in everyday practice both for women (fear of cancer) and for clinical and diagnostic experts.

Complementary methods used in revealing, observation and final diagnosis of secerning breast-clinical examination, mammography, ultrasound examination, cytodiagnosis, galactography, histopathology – have different characteristics and diagnostic possibilities, but behave as supplementary and contribute individually to setting the correct diagnosis of the disease.

In the case presented in this paper the only symptom of the disease – breast discharge – initiated several diagnostic procedures that discovered pathological lesions in the breast.

Cytological diagnostics of the discharge revealed the presence of intraductal papilloma and the patient underwent surgery. This was followed by histological examination of the breast tissue which not only pointed at the intraductal papilloma, but also at the tumor cells dispersed in the surrounding stroma. Although the cytological smear of the discharge was tumor cells negative (only the cells of intraductal papilloma were found), a surgical procedure which included pathohistological examination of the surrounding breast tissue was performed and individual tumor cells, hardly visible by clinical examination, were revealed. An extended surgical procedure was undertaken – a total mastectomy including axillary lymph nodes removal.

Pathohistological examination of the total breast tissue resulted in the finding of diffuse signet ring cells spread with no involved the regional lymph nodes.

In conclusion: intraductal papilloma could be the first symptom of the breast carcinoma and should require a serious approach.

## Competing interests

The authors declare that they have no competing interests.

## Authors' contributions

VB performed the histological examination of the breast tumor tissue, conceived of the study, participated in its design and coordination and was a major contributor in writing the manuscript. BS and MP performed cytological examination of material from a nipple discharge. JK performed total mastectomy, axillary limphadectomy and contra lateral breast biopsy. ZK performed ductulolobular segmentectomy. DŠ performed ultrasound examination. ZI performed mammography. ZI and IT carried out post-operative oncology therapy. MB performed imunohistochemistry and participated in reference research. All authors read and approved the final manuscript.

## Consent

Written informed consent was obtained from the patient for publication of this case report and accompanying images. A copy of written consent is available for review by the Editor-In-Chief of this journal.
